# Vertical Fracture of the Odontoid Process

**DOI:** 10.7759/cureus.20204

**Published:** 2021-12-06

**Authors:** Douglas J Quint, Suresh Ramnath

**Affiliations:** 1 Radiology, University of Michigan, Ann Arbor, USA; 2 Neurosurgery, University of Michigan, Ann Arbor, USA

**Keywords:** magnetic resonance imaging, neck, odontoid process, dens fracture, cervical spine

## Abstract

Fractures of the odontoid process of the axis usually occur transversely at the neck or base of the odontoid, are often displaced, and frequently require surgical fixation. Sagittal or coronal fractures are uncommon and can best be visualized on coronal or sagittal reconstruction of CT scans. Routine radiographs may not allow precise diagnosis. Vertical fractures, either sagittal or coronal, generally do not require operative treatment. This report describes an unusual fracture of the odontoid process sustained by a 56-year-old male after falling down a flight of stairs. He was neurologically intact, and the fracture healed with immobilization in a rigid cervical brace. Only 11 other case reports have been identified in a literature review. Both coronal and sagittal reconstructions should be obtained in suspected cases of odontoid fracture. Without instability on flexion/extension views or ligamentous injury on an MRI scan, a rigid brace or halo vest can be used to promote healing of the fracture, which may occur in 12 weeks.

## Introduction

Fractures of the odontoid process (dens) of the second cervical vertebra (axis) are usually transverse, at the junction of the dens and the body of the axis, or more cranially, at the level of the isthmus. Vertical fractures are uncommon and difficult to recognize on routine radiographs, instead requiring reconstructed views of a CT scan for accurate diagnosis. Most patients with vertical fractures are neurologically intact. This report illustrates a coronal vertical fracture of the odontoid process sustained in a fall, in a neurologically intact patient, successfully treated without operative intervention.

## Case presentation

A 56-year-old man fell down a flight of stairs while sleepwalking and lost consciousness for an unknown period, only regaining awareness in the hospital emergency department. Lacerations of the vertex and posterior scalp were sutured.

**Figure 1:**
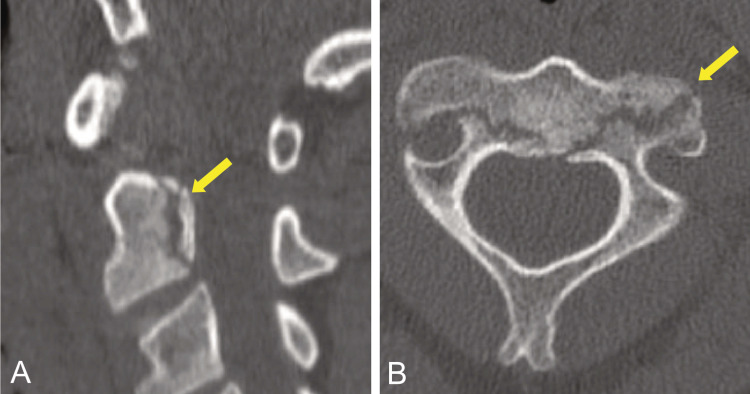
Computed tomography scans (A) Left paramidline (yellow arrow) and (B) fractures (yellow arrow) extending to the region of the foramina transversaria bilaterally.

**Figure 2:**
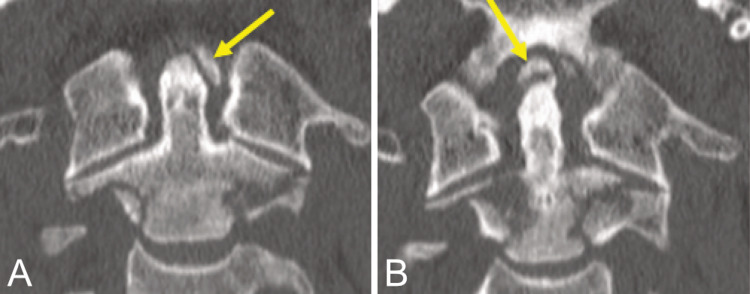
Computed tomography scans (A) Left alar ligament avulsion fracture (yellow arrow) and (B) apical ligament avulsion fracture (yellow arrow).

Work-up included CT scans of the brain and cervical spine. No significant intracranial abnormalities were identified. The cervical spine study demonstrated a vertical fracture of the axis beginning at the posterior aspect of the apex of the dens and descending into the body of the axis where it continued laterally into the right foramen transversarium, and just anterior to the left foramen transversarium (Figures 1A, 1B, 2). A small osseous fragment was identified just to the left of the apex of the dens, suggesting an avulsion fracture associated with the alar ligament (Figure 2A), and also an apical tip fragment suggestive of an apical ligament avulsion fracture (Figure 2B). There were also fractures of the posterior arch of the atlas just posterior to the lateral masses. The patient was neurologically intact and was discharged a few days later, wearing a rigid cervical brace.

The patient was then seen by our group a few weeks later, with ongoing neck pain and vertigo, but with an otherwise essentially normal neurologic examination. He continued to wear the brace, and on follow-up CT imaging approximately three months after the event demonstrated good healing of the fractures. He also reported improving neck pain.

## Discussion

**Figure 3:**
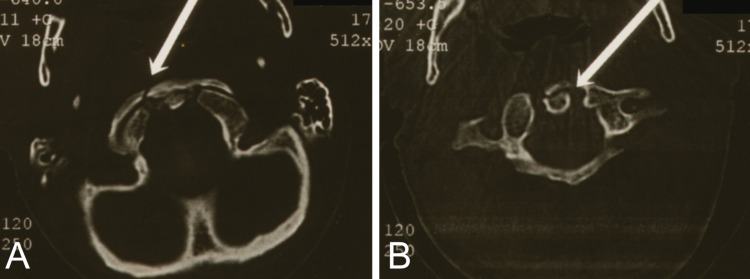
Computed tomography scans (A) Fracture of the anterior arch (white arrow) of the atlas and (B) coronal vertical fracture (white arrow) of the dens in a 76-year-old male.

A PubMed literature review revealed 11 other reports of vertical dens fractures, with only two occurring in the sagittal plane and the others occurring in the coronal plane. Ages ranged from 14 to 89 years; nine were males and two were females. The injuries were the result of motor vehicle accidents in seven patients and falls in three patients. In one instance, a large slab of ice fell from a roof striking the vertex of the patient’s head, and in another, a young man struck his head on a tree as he was diving for a football. Ten of these patients were neurologically intact at the time of injury. One other patient treated by coauthor S.R. was a 76-year-old male who fell and sustained a coronal vertical fracture of the dens, extending to the junction of the dens to the axis, and an associated fracture of the anterior arch of the atlas. He was neurologically intact and made an uneventful recovery after immobilization of the neck in a rigid brace for 12 weeks (Figures 3A, 3B).

The patient of Scott et al. [1] had sustained lethal injuries from atlanto-occipital and atlanto-axial dislocation, and at autopsy, there was a crush injury of the spinal cord at the medullo-spinal junction. One anteroposterior X-ray of the cervical spine showed a fracture from just inferior to the apex of the dens on the left extending obliquely to the junction of the dens and the body of the axis on the right, very similar to the fracture in the patient reported by Mete et al. [2].

Bergenheim and Forssell’s [3] patient also demonstrated bilateral frontal epidural hematomas and a separation of the coronal suture. The hematomas were drained, and the patient then developed paraparesis two days later, prompting imaging evaluation of the spine. The paraparesis in this patient was not likely related to the dens fracture, although injury to the crossing cortico-spinal fibers in the lower medulla could conceivably have caused this.

Althoff [4] classified fractures of the odontoid into four types. Type A is described as a fracture at the tip of the dens (also called type I by Anderson and D’Alonzo [5]). Benzel et al. [6] suggest this is essentially an avulsion of the attachment of the alar ligament taking with it a fragment of the dens. Althoff’s type B (called type II by Anderson and D’Alonzo [5]) is a fracture at the base of the dens. Althoff’s types C and D (called type III by Anderson and D’Alonzo [5]) involve the base of the odontoid process extending into the more rostral portion of the body of the axis and to one or both facets. Benzel et al. [6] further subdivide these latter types (Althoff C and D, or Anderson/D’Alonzo III) into vertical and horizontal fractures.

In our patient, and in the cases reported by Ben Amor et al. [7], Francavilla et al. [8], and one from Castillo and Mukherji’s series [9], the odontoid fracture extends into the body of the axis. In addition, three of these patients had associated fractures of the anterior or posterior arch of the atlas. One of Castillo and Mukherji’s patients also appears to have sustained a comminuted fracture of the odontoid tip [9].

Our patient shows a probable type I alar ligament avulsion fracture, a vertical fracture extending coronally as a type III fracture into the body of the axis bilaterally along with fractures of the lamina of the atlas. The scalp lacerations do not provide a satisfactory explanation for the combination of fractures, only suggesting that as he fell, he may have struck the top and back of his head against some steps with the head and neck extended. The cases of Ben Amor et al. [7], Bergenheim and Forssell [3], and Katoh et al. [10] sustained facial and anterior cranial injuries, which are more convincing for an extension force to the head. Bergenheim and Forssell [3] hypothesized that an axial load directed caudally combined with head extension caused a backward movement of the head and the anterior edge of the foramen magnum to strike the tip of the odontoid in the axial plane, acting like a chisel to split the dens. This does not explain the solitary sagittal fracture reported by Mete et al. [2] and Scott et al. [1]. The mechanism of injury should not disrupt the transverse ligament.

## Conclusions

In suspected fractures of the odontoid, both coronal and sagittal reconstructions should always be obtained. In only two of the reported cases, there was posterior displacement of the dens. In the absence of instability on flexion/extension views or ligamentous injury on an MRI scan, healing can occur with immobilization in a rigid brace after about 12 weeks, although a halo vest may also be used.
